# Early Life Vaccination of Companion Animal Pets

**DOI:** 10.3390/vaccines9020092

**Published:** 2021-01-27

**Authors:** W. Jean Dodds

**Affiliations:** Hemopet, 11561 Salinaz Avenue, Garden Grove, CA 92843, USA; info@hemopet.org; Tel.: +1-714-891-2022 (ext. 115); Fax: 714-891-2123

**Keywords:** early life innate and adaptive immunity, vaccines

## Abstract

Development of the immune system of mammalian animal species parallels that of humans and involves the innate and adaptive (acquired) immune responses acting together with the thymus gland. Consequently, issues surrounding the adequacy and safety of vaccinations to protect pet animals from their relevant infectious diseases need to be addressed just as they are for humans. Pet animals, especially canines, also have unique needs because of the wide diversity of purebred and mixed breeds that vary greatly in size, type, temperament, and even maturation rates. Furthermore, pets in early life encounter a series of changes that can affect their development and induce stressors including parasite control, new homes and environment, novel foods, and the socialization that is essential at a time when vaccinations need to be given. While recognizing that this overall need is becoming more understood, current vaccination policy guidelines for companion animals are still only adhered to by about 40% of veterinarians worldwide. Clearly, vaccination of pets should no longer be considered as “one size fits all”.

## 1. Introduction

The immune system of companion animal pets, like that of all mammals, forms the backbone of body function to ensure health, well-being, and longevity while protecting against the foreign invaders of disease [1,2,3,4,5,6,7,8,9,10,11,12,13,14,15,16,17,18]. Once the newborn’s innate and maternally derived immunity from colostrum milk wanes, the infant becomes susceptible to disease exposures until his own adaptive immunity and immune memory develops and becomes functional [1,2,5,6,7,8,12,13,14,15,17]. In between these two events, termed the “window of susceptibility”, the infant is at risk for succumbing to infectious diseases [11,12].

For literally hundreds of years, vaccinations have played a key role in offsetting the undeveloped adaptive immunity of the young human and animal [1,2,9,16]. Both the innate and adaptive (acquired) immune systems and immune memory evolve along with the functional roles of the pituitary–thyroid–hypothalamic axis, the thymus gland and its gradual involution, and the maturation of the microbiome in the gut [1,2,6,7,13,18,19,20,21,22,23].

A unique feature of the immune system is the induction of immune memory [1,12]. Memory cell immunity is created by bursal-dependent (B-cell) and thymus-dependent (T-lymphocytes) that respond to an immune stimulus, whether an infection or vaccination [1]. These cells replicate when responding to an invading antigen, form clones that retain information about each prior exposure, and thereby generate immune memory. Following this exposure, serum antibody levels begin to rise in about a week and peak around 10 days [1,12]. Antibody levels then decline but remain higher than in the naive unexposed state. If the same immune stimulus is encountered again, the immune system mounts a faster and more powerful anamnestic response, such as with viral exposures and vaccines [1,10,12]. While the immune system’s capacity for memory generates immunity through vaccines, it can also trigger adverse events such as autoimmune disorders and allergies/hypersensitivity [1,10,24,25,26,27,28,29,30]. Additionally, the relationship of the gut microbiome in the development and function of the immune system may ultimately affect vaccine efficacy as well [31].

### Early Life Immune Responses

Innate immune response is the ready to go “first responder” of immune protection. This immune system is composed of physical and chemical barriers, phagocytic leukocytes, dendritic cells, natural killer cells, and plasma proteins. It provides barriers to infection, while the circulating soluble factors of the complement system provide additional support [1,5,6,7,8,14,16,22].

Adaptive (acquired) immune response develops as memory immunity after a pathogen exposure such as bacteria, viruses, and fungi [1,2,6,13,14,15,17,18,24]. The innate and adaptive immune responses work together to eliminate pathogens from the body. There are two types of adaptive responses: the cell-mediated immune response, controlled by activated T-cells, and the humoral immune response, controlled by activated B-cells and antibodies. Adaptive immunity is defined by the presence of either T- or B-lymphocytes, and includes both CD8+ cytotoxic effector T-cells that directly destroy tumor cells, CD4+ helper T-cells that regulate CD8+ T-cell and B-cell function, and B-cells that present antigen and produce antibodies. The regulatory and effector molecules of this adaptive immune response sustain the antigen–antibody binding functions that release effector cytokines (small cell signaling molecules) and their specific family of chemokines that direct chemotaxis and attract cells to sites of inflammation and infection. The important cytokines include the chemokines, interferons, interleukins, and tumor necrosis factor alpha (TNF-α) [1,2,3,4,5,6,7,14].

Equally important for this evolution of early life immune response is the protective effect of maternally derived immunity in neonatal dogs and cats and fetal mice and humans [5,6,7,8,12].

The thymus is a primary lymphoid organ that plays a vital role in the induction of central immune tolerance and the development of T-cells [19,20,21]. T-lymphocytes also orchestrate the development of the adaptive immune response [1,5,6,7]. In many species, except dogs, cats, and mice, the thymus starts decreasing in size at birth, although the immunosenescence associated with a decline in thymic output is an evolutionary conserved event [19,20,21,22,23,24,25,26]. Furthermore, the thymus gland can involute transiently with stress, infections, pregnancy, and malnutrition [20].

In dogs, age-associated changes in thymus size and output vary with the longevity of dog breeds [21]. For example, smaller breeds that live longer than large and giant breeds have varying amounts of growth hormone, insulin-like growth factor-1 (IGF-1), and interleukin -7 (IL-7) that influence T-cell development and their rate of aging. Interestingly, no sex differences have been identified with thymus function and development [19,21].

## 2. Vaccine Issues Revisited

### 2.1. Summary on Vaccine Policy

American Animal Hospital Association (AAHA) 2003—Current knowledge supports the statement that [26]:

- No vaccine is always safe, no vaccine is always protective and no vaccine is always indicated.
- Misunderstanding, misinformation and the conservative nature of our profession have largely slowed adoption of protocols advocating decreased frequency of vaccination.

World Small Animal Veterinary Association (WSAVA) 2015–2017—From the late Prof. Michael J. Day [27,28]:

- Vaccination should be just one part of a holistic preventive healthcare program for pets that is most simply delivered within the framework of an annual health check consultation.
- Vaccination is an act of veterinary science that should be considered as individualized medicine, tailored for the needs of the individual pet, and delivered as one part of a preventive medicine program in an annual health check visit.

#### 2.1.1. Vaccination May Not Result in Immunization

Importantly, pet caregivers should understand that the act of giving a vaccine may not equate to immunization of that animal [11,12,25,26,27,28,29,30,31]. Vaccines may not always produce the needed or desired immune protective response, not only if the vaccine itself was inadequately prepared (exceedingly rare) but also if the pet is a genetic low or non-responder to that vaccine (may be common in certain breeds of dogs and their families) [2,4,9,10,11,12,13,14,15,16,17,18,24,25]. In the latter case, that pet will be susceptible lifelong to the disease of concern and revaccination will not help and could even be harmful [9,10,11,12].

#### 2.1.2. New Protocols Based on Vaccine Policy Guidelines

In response to the issues raised above, vaccine experts recently have recommended new protocols for dogs and cats [11,27,28,29]. These include: (1) giving the puppy or kitten vaccine series later (starting not before 8 weeks of age, except in the cases of outbreaks of virulent viral disease, in orphans, or those that never received colostrum from their dams) followed by a booster at one year of age; (2) administering further boosters in a combination vaccine every three years or as split components alternating every other year until; (3) the pet reaches geriatric age, at which time booster vaccination is likely to be unnecessary (except when legally required), and can be unsafe for those with aging-related or immunologic disorders.

#### 2.1.3. Alternatives to Vaccine Boosters

In the intervening years between booster vaccinations, and in the case of geriatric pets, circulating humoral immunity can be evaluated by measuring serum vaccine antibody titers as an indication of the presence of immune memory [10,11,25,26,27,28,29,32,33,34,35,36,37,38,39,40]. Titers do not distinguish between immunity generated by vaccination and/or exposure to the disease, although the magnitude of immunity produced just by vaccination is usually lower [11,25,26,27,28,29].

#### 2.1.4. Role of Passive and Neonatal Immunity

Passively derived maternal immunity hampers active immunization of newborns [5,9,11,12,22,25,26,27,28,29]. Furthermore, an immature immune system contributes to a weak and T-helper cell type -2 (Th2) polarized immunity [1,4,5,6,7]. This state of immunity in early life sustains endemic infections in humans and continuous reinfections in animal herds [5,6,7,8,34]. The endemic infections of the young occur preferentially when the immune system is still functionally immature and when the low levels of maternal antibodies are no longer protective, but yet block protective immune responses [1,2,4,5,6,7,8]. The Th2 bias of the newborn is mediated by high levels of progesterone and Th2 cytokines produced in the maternal–fetal interface [8,22].

The innate immune system’s activity is enhanced in the mother during the prepartum period, and this can have both beneficial and potentially harmful effects on the offspring [5,6,7,8,9,10]. Viral exposures at a young age are important to promote proper immune system development [13,14,15,16,17,18]. In addition, the immune system is continuously stimulated by systemic commensal viruses at low levels sufficient to develop resistance to other infections. Additionally, newborns can have their immune systems primed by exposure to innocuous environmental antigens, such as non-pathogenic bacteriophages, and noroviruses in humans [13,18]. This has a strong lifelong effect with subsequent exposure boosts. Importantly, this priming effect can be modulated by adjuvants that focus on modulating T-helper cell type -1 (Th1) and Th2 [2,6,7,8,9,10,41]. In various species, including mice, dog, sheep, horse, and seal, a strong Th1 stimulating adjuvant can induce immune responsiveness and protection in newborns, even when conventional vaccines fail [6].

#### 2.1.5. Clinical Utilization of Vaccine Policy

Veterinarians and pet caregivers realize and should always keep in mind that the purpose of appropriate early life immunization is to prevent and protect these animals from vaccinable infectious diseases [11,27,28,29,30]. Thus, the clinical efficacy and safety of vaccination protocols are essential in achieving this goal [11,27,28]. As discussed below, not all vaccines are considered to be essential, and the need for optional, non-essential vaccines depends upon the geographical exposure risk and lifestyle of the pet and caregiver [11,27,28]. Local veterinary groups and public health agencies can offer up-to-dare data listings of the area’s zoonotic and infectious disease cases in companion animal dogs and cats [27].

#### 2.1.6. Role of Governmental Regulatory Oversight

The United States Department of Agriculture (USDA) is tasked with considering and implementing appropriate regulatory steps based upon the evolving technologies, individual and herd animal needs as well as societal, commercial, and stakeholder perspectives [42]. The agency’s December 2020 report represents an update of the recent additions and changes with regard to veterinary vaccines. A second goal of the report was to increase the transparency of veterinary adverse reaction reports and describe the current efficacy and safety standards required by the USDA for product licensure [42]. This report is especially timely given the global impact of the current COVID-19 pandemic, and the increasing number of vaccine skeptics and naysayers throughout the world [43].

### 2.2. Some Beneficial Effects of Vaccines

#### 2.2.1. A Widely Recognized World Invention

Vaccines comprise one of the world’s most recognized inventions that began centuries ago in around 1000 A.D. with the practice in China and India (termed “variolation”) [9]. Today, this original approach of variolation has evolved to become modern vaccination practices, which are now mostly standardized worldwide [9,10,27,28,29]. Vaccination helps to protect humans, companion animals, livestock, wildlife, and birds from their respective common infectious diseases [1,9,10,11,27,28,29,44,45].

#### 2.2.2. Role of Modern Vaccine Technology

While modern vaccine technology has afforded effective protection of companion animals against serious infectious diseases, this advancement brings the increased risk of rare but real potential for adverse reactions (termed “vaccinosis”) [1,10,11,27,28,29,46,47,48,49,50,51,52,53,54,55,56]. Some adverse events are serious, chronically debilitating, and even fatal [11,27,28,29]. Thus, we must balance this benefit–risk equation. As Professor Ronald Schultz has stated, “Be wise and immunize, but immunize wisely!”

#### 2.2.3. Saving Countless Human and Animal Lives

More human and animal lives have been saved by vaccinations than with any other medical advance. Furthermore, vaccines have permitted more animal agricultural production and wildlife species’ biodiversity to be safeguarded [1,11,27,28,44].

#### 2.2.4. Serious Infectious Diseases Controlled or Eradicated

The world eradicated smallpox in 1979, and nearly all polio and measles in people has been, except for the recent recrudescence of mild polio symptoms in some elderly people vaccinated decades ago.

The first vaccines were against smallpox and anthrax, and canine distemper, first recognized in the 1600s as a neurological disease of dogs and now known to be caused by the canine distemper virus [11,12].

Vaccination has significantly reduced endemics of canine distemper, hepatitis, and parvovirus in domestic pets, but is still present in wildlife reservoirs of Canidae species [10,44].

The risk of endemic feline panleukopenia virus has significantly abated with the long duration of immunity conveyed by the current vaccines [25,28].

Rabies vaccination has essentially eliminated rabies in Europe; vaccination has eradicated Rinderpest in Africa, and foot and mouth disease in Europe [1,40,46].

### 2.3. Some Harmful Effects of Vaccines

#### 2.3.1. Acute Postvaccinal

Events include: immediate hypersensitivity reactions, other acute events that tend to occur 24–72 h or up to one to two weeks afterwards [10,11,46,47]. In the case of more delayed reactions, these can occur up to 45 days and even 60 days later, especially with rabies vaccines containing thimerosal (mercury) [9,10,11,40,41,42,43,44,45,46,47,48,49,50,51,52,53,54,55,56,57]. These reactions are seriously under-reported to the manufacturers and government regulatory authorities [1,9,42,58,59,60,61,62].

In cats, while adverse vaccine reactions may be less common, aggressive tumors (fibrosarcomas) can occasionally arise at the site of vaccination, as they can in dogs [11,28,29,54]. Other cancers such as feline leukemia have been vaccine-associated [54].

Postvaccinal polyneuropathy is a recognized entity associated occasionally with the use of mostly canine distemper, rabies, and ovine bluetongue virus vaccines [11,30,63,64,65]. Clinical signs include: muscular atrophy and excitation, incoordination and weakness, separation anxiety, destruction and shredding of objects, unprovoked aggression, obsessiveness, barking, fearfulness, self-mutilation, tail chewing, pica, and seizures [11,30].

#### 2.3.2. Genetic Predisposition to Adverse Vaccine Events

Certain breeds or families of dogs are more susceptible to adverse vaccine reactions, exhibited as postvaccinal seizures, high fevers, and painful episodes of hypertrophic osteodystrophy (HOD). Therefore, we need to advise companion animal breeders and caregivers of the potential for littermates and relatives to be at increased risk for similar events [11,26,30]. The recently weaned young puppy or kitten being placed in a new environment may be at particular risk. Furthermore, while the frequency of vaccinations is recommended to be spaced 2–4 weeks apart, some veterinarians advocate giving vaccines once a week in perceived or legitimate high exposure risk situations [10,11,27]. This practice makes little sense scientifically or medically, and clearly can be harmful.

#### 2.3.3. Vaccine Dosage

All dogs, no matter what size or breed type, are currently vaccinated with the same quantity of vaccine [27,28,29]. Yet, why do very small and very large dogs need the same dose of vaccines, when the manufacturer’s vaccine clinical trials are typically performed on laboratory beagles and with minimal field testing in different breed types prior to licensing [10,11]?

More vaccine adverse events have been documented in smaller dogs [11,30,55]. Toy and smaller dogs logically should require less vaccine than giant and large dogs to be fully immunized [11,55]. Similarly, puppies and kittens should require less vaccine volume to immunize than adults. In support of the size hypothesis, this author studied healthy adult, small breed dogs that had not been vaccinated for at least three years. They were given a half-dose of bivalent canine distemper and parvovirus (DPV) vaccine, whereby all of them developed increased and sustained serum vaccine antibody titers [57].

#### 2.3.4. Vaccine Overdosing

Giving vaccines to pets too early in life and too frequently not only can be ineffective if partially or mostly neutralized by residual maternal immunity, but also presents the risk of inducing adverse effects [2,11,27,28,29]. Other issues with over-vaccination include increased costs in time and dollars spent, despite the well-intentioned solicitation of clients so that pets can also receive a wellness examination. Giving unnecessary annual boosters has the client paying for services that likely are of little benefit to the pet’s existing level of protection against these infectious diseases. It also increases the risk of adverse reactions from the repeated exposure to foreign substances [1,2,10,11,16].

#### 2.3.5. Adjuvants

Adjuvants act to accelerate, prolong, or enhance antigen-specific immune responses [16,41,55,58,59]. They are added into vaccines to enhance their immunogenicity, but this increases risk of autoimmune and inflammatory adverse events following vaccination [41,51,55,58,59]. For the killed vaccines available for human and veterinary use, potent adjuvants and additives are included to produce a more sustained humoral immune response and compete favorably with the longer protection typically afforded by MLV products. While current vaccine adjuvants can successfully generate humoral antibody-mediated protection, other diseases such as tuberculosis and malaria require a cell-mediated immune response for adequate protection. Importantly, while killed, inactivated vaccines containing adjuvants make up only about 15% of veterinary biologicals used, they have been associated with 85% of the post vaccination reactions [11,26,27,28,41,48,49,50,51,52,53,54,55]. However, although adjuvants have been used safely in human and veterinary medicine for decades, and millions of doses have been given with relatively few documented adverse events, there is increasing worldwide concern about the safety of using heavy metals such as thimerosal (mercury) and aluminum [16,41,48,55,56,57,58,59,60,61]. Vaccine pharmacovigilance is the term used to remind us all that vaccines carry an inherent risk, albeit small [41]. Vigorous opposition to this belief is also ongoing [62].

#### 2.3.6. Presence of Ultra-Trace Metals

Vaccinations have helped immensely to diminish the impact and spread of disease, although recent attention had been focused on the role of and potential harm from including heavy metals as vaccine adjuvants in killed inactivated vaccines [41,47,48,49,50,51,52,55]. The addition of aluminum and mercury in adjuvants for these vaccines is intended to boost the antigen-specific immune responses to make the vaccines work more quickly or effectively. Of concern, however, is the fact that these adjuvants can cross the blood–brain barrier and remain there indefinitely [55]. When added to the expected low level of dietary ingestion, these metals can then accumulate in the body and can lead to liver damage, gastrointestinal inflammation, kidney damage, and brain toxicity. Ultra-trace minerals, including chromium, nickel, molybdenum, silica, and aluminum, are not regulated by the National Research Council (NRC) or the American Association of Feed Control Officials (AAFCO), and there are currently no set safe upper limits, especially for dry pet foods [55]. Except for aluminum which has no known nutritional role, other minerals do play a role in metabolism and have specific dietary requirements. These findings indicate that the potential for adverse effects following exposure to and accumulation of heavy metals in vaccinations and other sources merits further investigation [55].

Additionally, infants and young children throughout the world receive a series of inoculations that include high quantities of mercury and now, aluminum. Incremental changes to the recommended vaccination schedules, along with the introduction of new aluminum-containing vaccines for pneumococcus and influenza, have significantly increased the quantity of metals in childhood immunizations, despite the federal U.S. phase-out of the use of mercury-based vaccine adjuvants between 2000 and 2002 [6,49,50,51,52,53,54,55]. Clearly, there is an urgent need to remove heavy metals from all vaccines, especially from those given to human and animal neonates and infants [49,50,51,52,55]. A safer alternative, calcium phosphate, has been approved by the World Health Organization and is naturally present in the body. Future vaccines will hopefully utilize calcium phosphate or other, safer alternatives to aluminum or other metals [55,59].

### 2.4. Compliance or Resistance to Current Vaccine Guidelines

While the issues discussed above about vaccines have been legitimately raised for over two decades, why is this knowledge still considered controversial [11,27,28,29,42,53,55,56,57,58,59,60,61,62,63,64]? Veterinarians and the public need to follow the national and international policies on vaccination guidelines [27,28,29]. Is it a matter of public distrust of the veterinarian’s role in earning income from promoting annual boosters or unnecessary vaccinations during “wellness visits” [42,43,61,62]? Admittedly, some veterinarians continue to assure their clients that there is no scientific evidence linking vaccinations with adverse effects and serious illness; this fallacy can confuse an impressionable client. On the other hand, vaccine and antivaccine zealots abound with hysteria and misinformation [11,43,62]. Neither of these polarized views are helpful. Given that clinicians may not understand the principles of vaccinal immunity, it is perhaps not surprising that attempts to change vaccines and vaccination programs for pets and people have created an ongoing debate and controversy [43,61,62].

### 2.5. Treatment of Vaccinosis

As the diagnosis of vaccinosis is an exclusionary one, nothing or minimal changes typically will be found to explain the patient’s symptoms [11]. The recently vaccinated animal that is unwell can be given the very safe oral homeopathics Thuja (for all vaccines other than rabies) and Lyssin to alleviate the symptoms of a vaccine-induced rabies miasm (molecular energy). This therapy should be given even if the clinical symptoms are actually unrelated to the recent vaccination. Therapy also often includes modest doses of steroids in tapering doses over 4–6 weeks to help stop the inflammatory process and clinical symptoms [11].

These patients should not receive further vaccine boosters, as serum antibody titers can be run instead to document protection. In the case of the rabies vaccine, exemption should be sought on a case-by-case basis but may not be granted in some locales [40,46].

### 2.6. Alternatives to Current Pet Vaccine Practices

Appropriate alternative options to current pet vaccine practices include: measuring serum antibody titers; avoiding unnecessary vaccines or over-vaccinating; deferring vaccinations of sick or febrile individuals; tailoring specific minimal vaccination protocol for dogs of breed types or families known to be at increased risk for vaccinosis; starting vaccination series later, when the immune system is more robust; alerting caregivers to watch their pet’s behavior and overall health after boosters; and avoiding revaccination of individuals that already experienced a significant vaccinal adverse event [11].

When an adequate immune memory has already been established, there is little reason to introduce unnecessary antigen, adjuvant and other excipients, as well as preservatives by administering booster vaccines [9,10,11,12,25,27,28,29,30]. By measuring serum antibody titers triennially or more often if needed, one can assess whether a given animal’s humoral immune response has fallen below levels of adequate immune memory [11,12,25,32,33,34,35,36,37,38,39,55]. In that event, an appropriate vaccine booster can be administered.

#### 2.6.1. Serum Antibody Titer Testing

Some veterinarians have challenged the validity of using vaccine titer testing to assess the immunologic status of animals against the common, clinically important infectious diseases [11,27,28,29,30,32,33,34,35,36,37,38,39,55]. This situation represents a misunderstanding of the so-called “fallacy of titer testing” because research has shown that once an animal’s titer stabilizes, it is likely to remain constant for many years [25,32]. Properly immunized animals have sterilizing immunity that not only prevents clinical disease but also prevents infection, and only the presence of antibody promoted by immune memory can prevent infection. As stated by eminent expert Professor Ronald Schultz in discussing the value of vaccine titer testing, these tests “show that an animal with a positive test has sterilizing immunity and should be protected from infection”. If that animal were vaccinated, it would not respond with a significant increase in antibody titer, but may develop a hypersensitivity to vaccine components (e.g., fetal bovine serum). Furthermore, the animal does not need to be revaccinated and should not be revaccinated since the vaccine could cause an adverse reaction (hypersensitivity disorder). You should avoid vaccinating animals that are already protected. It is often said that the antibody level detected is “only a snapshot in time”. That is simply not true; it is more a “motion picture that plays for years”.

However, not all vaccines produce sterilizing immunity. Those that do include: canine distemper virus, canine adenovirus, and canine parvovirus, and feline panleukopenia virus (a parvovirus of cats). Vaccines that produced non-sterile immunity would be leptospirosis, *Bordetella*, rabies virus, herpesvirus and calicivirus—the latter two being upper respiratory viruses of cats [28]. While non-sterile immunity may not protect the animal from infection, it should keep the infection from progressing to severe clinical disease [11,12,27,28,29].

#### 2.6.2. Protection against Disease with Sterile and Non-Sterile Immunity

For vaccines that elicit sterile immunity, the presence of any measurable antibody indicates protection. While a positive titer test result is straightforward, a negative titer test result is more difficult to interpret, because a negative titer is not the same thing as a zero titer and it does not necessarily mean that the animal is unprotected. A negative result usually means the titer has failed to reach the threshold of providing sterile immunity. This is an important distinction, because it may mean that the animal is unprotected against these clinically important diseases [27,28,29].

Regardless of any dispute, several decades of experience with vaccine titer testing reveals that 90–98% of dogs and cats that have been properly vaccinated develop good measurable antibody titers to the infectious agent measured [27,28,29,30,32,33,34,35,36,37,38,39].

With regards to rabies virus, results of the Rabies Challenge Fund research study published in early 2020 showed that [46]: (i) duration of immunity to rabies in vaccinated dogs extends beyond 3 years; (ii) immunologic memory exists even in vaccinated dogs with serum antibody titer 0.1 IU/mL; and (iii) non-adjuvanted recombinant rabies vaccine induces excellent antibody responses in previously vaccinated dogs 14 days after administration.

## 3. The Author’s Preferred Vaccination Protocol

The author’s rationale for selecting this small animal vaccination protocol is based upon several factors, including: incorporating vaccines for those common infectious disease agents that are known to cause significant clinical illnesses or death in dogs and cats. (these are the vaccines designated as “Core” by the world’s veterinary policy advisors); the age at which it is considered safe to give MLV, recombinant or inactivated vaccines; and the age when most if not all of the residual maternal immunity has waned in order to ensure efficacious immunization. Furthermore, other vaccines are designated as optional and are recommended based upon the exposure and lifestyle risk for pets in the animal’s geographical locations. Lastly, these recommendations include the safety of recent vaccinations to leave their home environment for socialization (Table 1).

### 3.1. Safety and Socialization 

#### 3.1.1. Puppies

- Three or more days after the last round of puppy vaccines, they can be out and about to be socialized. In the interim period, between 10 and 14 weeks of age, socialization can take place in the back yard or at puppy training classes with known friends and healthy dogs. [29,30,64]
- Until fully vaccinated, puppies should not walk on unfamiliar or public grounds; they can be carried about, when needed to travel.
- If titer testing is desired, instead of giving another vaccine after 12 weeks of age, wait until at least 16 weeks of age to avoid measuring residual maternal immunity.

#### 3.1.2. Kittens

- Socialization and vaccine titer testing options are as for puppies.

## 4. Discussion

The first scientifically proven smallpox vaccine was developed by Edward Jenner using cox pox as the inoculum in 1796 [66]. Even before that, this approach was used privately by Benjamin Jesty in 1774 to protect his family from smallpox [10,66]. Since then, for nearly 100 years, vaccines have been used commercially and for research in humans and animals to elicit protective immunity to specific infectious agents [10,11,27,28,40,41,58,59,60,61,62,63,64,65]. Despite these advances in vaccine-related issues, this topic continues to be one of the most contentious of all human and animal medical safety and efficacy procedures [24,27,58,59,60,61,62]. Regardless, new approaches and research tools are still needed to effect worldwide protection from current and emerging infectious diseases not only for humans, but also for companion animals, pocket pets, birds, laboratory animals, livestock, and wildlife [9,10,11,31,44,63,64,65,66,67,68,69,70,71].

There is little doubt that the application of modern vaccine technology has permitted us to effectively protect companion animals and people against serious infectious diseases [11,27,28]. However, vaccinations are increasingly recognized contributors, albeit relatively rare, to immune-mediated blood, skin, bowel, bone, and joint diseases; bone marrow and organ failure; central nervous system excitation; and behavioral aberrations [10,11,44,59,60]. Genetic predisposition to these adverse vaccinosis events has been documented [11,24,30]. It must be recognized, however, that we have the luxury of expressing these concerns today only because the risk of disease has been effectively reduced by the widespread use of vaccination programs [9,10,11].

Several factors are known to contribute to the risk of adverse vaccine reactions, namely: genetic predisposition (family history and breed type), age and size, influence of sex hormonal change (estrus), and type of vaccine and adjuvant used (rabies and thimerosal, bluetongue virus and aluminum salts) [10,11,18,26,27,28,29,49,50,51,52,53,54,55].

An augmented immune response to vaccination is seen in dogs with pre-existing inhalant allergies (atopy) to pollens [24]. Furthermore, the increasing problems with allergic and immunological diseases have been linked to the introduction of MLV vaccines more than 20 years ago [9,10,11,45]. The accumulated evidence indicates that vaccination protocols should no longer be considered as a “one size fits all” program [10,27,28,29,56].

For veterinary medicine, annual vaccination has been and remains the single most important reason why most owners bring their pets for an annual “wellness visit” [11,27,28]. Reluctance to change current vaccination programs is fueled by the lack of understanding of the principles of applied vaccinal immunity, which is rarely taught even at the postgraduate level [11,12,13,14,16,25,26,27,28]. Today, only an estimated 40% of veterinarians follow the latest vaccine policy guidelines, as they have no regulatory authority [27,28,29]. Many pet caregivers receive written or telephone reminders that their pet is “due” for vaccinations or is “up-to-date” on vaccinations [29].

The debate and controversies surrounding human and animal vaccines and vaccination policy will not be resolved in the foreseeable future [11,31]. The collective experience of the scientific community depends upon the development and implementation of new technologies [9,67,68,69,70,71].

### 4.1. Efficacy Issues

Before being licensed, all commercial vaccines are tested first in experimental animals (e.g., rodents, rabbits, guinea pigs) and then, in clinical trials typically done in Phases 1, II, III, and IV for humans, and experimental species-targeted animals followed by clinical trials in the intended species in the case of veterinary vaccines [10,11,27,41,70]. One novel approach used electron-microscopy to determine if there were solid particulate contaminants in vaccines [9]. Micro- and nano-sized particulates composed of inorganic elements were found in 43 of 44 vaccines studied, despite the fact that they were not declared among the components. The only vaccine without these particles was a feline 3-way vaccine; all the others were human vaccines made by a variety of manufacturers and for various diseases, including those for typhoid, tetanus, diphtheria, pertussis, hepatitis B, polio, Hemophilus influenza, measles, mumps and rubella, chicken pox, yellow fever, pneumococcus, and meningococcus [9].

A more recent concept, termed “reverse vaccinology”, shows promise as a promising candidate computer-based approach of vaccine development to induce innate, non-specific immunity for long periods [71]. It identifies candidate vaccine antigens and is highly sensitive, although the technique is not specific and not hypothesis driven. To date, it has been used in studies of meningococci and tuberculosis organisms which were made possible once whole genome sequencing technology was available. The future intent is to replace today’s vaccine adjuvants and non-toxic derivatives of toxins with “trained-immunity based vaccines” [71].

### 4.2. Modified-Live versus Killed, Inactivated Vaccines

The late Dr. Jonas Salk indicated that killed inactivated vaccines are always preferable to those of modified live virus origin because they are safer [11,45]. The hotly debated costs, convenience, and risks concerning live (Sabin) versus inactivated (Salk) human polio vaccines is one example. As stated above, 10% of licensed and used veterinary biologicals are of the killed, inactivated type and yet they account for 85% of the reported postvaccinal adverse reactions [41]. This discordancy is thought to be caused by the effects of various adjuvants and other excipients (cell tissue culture remnants, egg protein, fetal calf serum, human and bovine serum albumin, yeast proteins, squalene, formaldehyde, antibiotics like neomycin and gentamicin, and other proprietary additives) used in vaccines for humans and other species [9,45,49,50,51,52,55,58,59,60]. What truly is “acceptable harm” in vaccine pharmacovigilance? As vaccines are generally viewed by the medical community and public as inherently safe, toxicity studies do not have to be included in regulatory safety assessments [41].

### 4.3. Are Vaccines Innocuous?

Vaccines clearly are not innocuous products and reported adverse events should no longer be denied [10,24,30,49,50,51,52,53,54]. Thus, the benefit/risk equation needs to be assessed in each individual before vaccination. The side effects of vaccines appear to be increasing lately in frequency and severity, particularly in children and in young pets [10,11,24,27]. The diphtheria-tetanus-pertussis vaccine has been linked to cases of sudden infant death syndrome; measles-mumps-rubella vaccine with autism; high-titer measles vaccines with childhood mortality; multiple immunizations with immune disorders; hepatitis B vaccines with multiple sclerosis, and the recent serious local or systemic adverse effects from the human papillomavirus vaccine [11,24,71].

### 4.4. Reducing Exposure Risk

The epidemiological goal of disease control is to reduce the exposure risk of susceptible human and animal populations to known infectious agents [10]. The typically recommended annual vaccine boosters for pet animals are not necessary and may be unwise in most cases, since the clinically important so-called “core” vaccines have a much longer duration of immunity than previously thought [11,27]. Boosters should be given only when absolutely necessary (such as with inadequate serum titer immunity to a “core” vaccine).

Non-adjuvanted, recombinant, subunit, synthetic vaccines (or the DNA/RNA vaccines under development) should be used whenever possible [11,27,67]. However, all rabies vaccines given to animals, as well as vaccines for canine leptospirosis, Lyme, canine influenza, and the injectable form of *Bordetella* are killed adjuvanted vaccines [27,41,47,63]. Cats have more vaccine options in comparison to dogs. For example, a non-adjuvanted feline rabies vaccine is available [40,46].

Studies to improve vaccinal immunity generally have paid little attention to the immune competence and efficacy of the host’s response to the immune challenge [10]. A recent study in dogs addressed the potential immune modulating effect(s) of stimulating specific acupuncture points along the body’s meridian system, GV-14, as practiced in Traditional Chinese Medicine [70]. This randomized trial used canine distemper virus (CDV) vaccine in 100 healthy client-owned dogs, ages 1–10 years, and quantitated the immune response after vaccination in both control and acupuncture groups. No significant differences were found between groups in age, weight, or sex, and both groups had highly significant increases in CDV serum neutralization titer post vaccination. The mean serum titer increase in the acupuncture group, however, was significantly greater than that of the control group. Thus, acupoint vaccination has the potential to enhance the immune response to this immunological challenge [70].

## 5. Summary

The need for vaccinations and necessary vaccines given within animal wellness checkups for companion animal pets should be discussed. Clients should understand that these vaccines are given to protect pets from prevalent infectious disease in their location.

However, a more pro-active approach is still needed to standardize and individualize the production and use of veterinary vaccines in order to ensure their safety and efficacy. Progress towards this goal remains hampered, however, by the ongoing controversy surrounding vaccines, failure to comply with current national vaccine policies and guidelines, resistance to change, and denial of adverse events within the general veterinary community as well as within society as a whole. The solution depends upon more focused educational efforts both within academic veterinary medicine, clinical practice, and the companion animal and livestock owner communities.

## 6. Conclusions

For the last 100 years, vaccines have proven their importance in providing protective immunity for human and animal populations. Despite these earlier, current, and new advances in vaccination development and technology, adverse events still occur in a small cohort of vaccinations. This had led to an ongoing worldwide contentious debate that is not likely to be resolved in the foreseeable future.

Veterinary practitioners are confronting increasing numbers of patients exhibiting signs of immunologic dysfunction and disease. The onset typically occurs within 30–45 days of vaccination. Evidence implicates vaccines and their adjuvants as potential triggering agents combined with genetic predisposition of the vaccinated host. The number of adjuvants used in veterinary vaccines should be re-examined. Discovery and implementation of new types of vehicles that enhance immune response to vaccines should be encouraged. A multifaceted approach is needed to recognize the situation, develop alternative strategies for containing infectious disease, and reduce the environmental impact of conventional vaccines.

Individuals that experience these adverse events are believed to be genetically predisposed rather than have reactions that are unexpected and idiosyncratic. To some veterinarians, canine and feline vaccination programs have been “practice management tools” rather than medical procedures. It is not surprising, therefore, that attempts to change vaccination programs based on scientific information have created significant controversy, and a “more is better” philosophy still prevails with regard to pet vaccines.

The adjuvants added to killed, inactivated vaccines are intended to enhance their degree and duration of immunogenicity in order to compete favorably with the typical longer immunity induced by modified live virus vaccines. These adjuvants generate a more robust and sustained humoral antibody-mediated immune response to many viral and other infectious agents.

To begin with, the periodicity between adult booster vaccinations should be increased to three or more years with monitoring of serum antibody levels (to assess protection against the clinically important infectious agents).

## Figures and Tables

**Table 1:** Author’s Vaccine Protocol.

Species	Age to Vaccinate (Weeks)	Vaccines	Booster or Serum Antibody Titer
		“CORE”	Except for rabies—1 year booster after completion of series, or measure serum antibody titers. Thereafter, booster or titer tests every 3 years.
*Puppies*	9–10 and 14–16, *or*	CDV, CPV, CAV-2 *	
	9, 12, and 16–18		
	20–24	Rabies virus	Rabies, 1 year booster, with 3 year boosters thereafter as legally required.
*Kittens*	7–9 and 12–16, *or*	FPV, FCV, FRT **	
	7, 11, and 16		
	18–24	Rabies, where legally required	Same as above.
		*Optional for Local* *Exposure Risk*	1 year boosters given, or measure serum titers.
*Puppies*	Separated from the above by 10–14 days	Bordetella, kennel cough, leptospirosis, canine influenza	
*Kittens*	Separated from the above by 10–14 days	FeLV, FIV, FIP, ꭞ Chlamydia	

* CDV—canine distemper virus; CPV—canine parvovirus; CAV-2—canine adenovirus (hepatitis). ** FPV—feline panleukopenia; FCV—feline calicivirus; FRT—feline rhinotracheitis virus. ꭞ FeLV—feline leukemia virus; FIV—feline immunodeficiency virus; FIP—feline infectious peritonitis virus.
